# Multivariate gene expression‐based survival predictor model in esophageal adenocarcinoma

**DOI:** 10.1111/1759-7714.13626

**Published:** 2020-09-01

**Authors:** Maoyuan Zhao, Jingsong Wang, Meng Yuan, Zeliang Ma, Yongxin Bao, Zhouguang Hui

**Affiliations:** ^1^ Department of Radiation Oncology, National Cancer Center/ National Clinical Research Center for Cancer/ Cancer Hospital Chinese Academy of Medical Sciences & Peking Union Medical College Beijing China; ^2^ State Key Laboratory of Molecular Oncology Chinese Academy of Medical Sciences & Peking Union Medical College Beijing China; ^3^ Department of VIP Medical Services, National Cancer Center/ National Clinical Research Center for Cancer/ Cancer Hospital Chinese Academy of Medical Sciences & Peking Union Medical College Beijing China

**Keywords:** Cox analysis, differential expression genes, esophageal adenocarcinoma, functional enrichment analysis, Kaplan–Meier analysis

## Abstract

**Background:**

Despite the recent development of molecular‐targeted treatment and immunotherapy, survival of patients with esophageal adenocarcinoma (EAC) with poor prognosis is still poor due to lack of an effective biomarker. In this study, we aimed to explore the ceRNA and construct a multivariate gene expression predictor model using data from The Cancer Genome Atlas (TCGA) to predict the prognosis of EAC patients.

**Methods:**

We conducted differential expression analysis using mRNA, miRNA and lncRNA transciptome data from EAC and normal patients as well as corresponding clinical information from TCGA database, and gene ontology (GO) and Kyoto Encyclopedia of Genes and Genomes (KEGG) pathway analysis of those unique differentially expressed mRNAs using the Integrate Discovery Database (DAVID) database. We then constructed the lncRNA‐miRNA‐mRNA competing endogenous RNA (ceRNA) network of EAC and used Cox proportional hazard analysis to generate a multivariate gene expression predictor model. We finally performed survival analysis to determine the effect of differentially expressed mRNA on patients' overall survival and discover the hub gene.

**Results:**

We identified a total of 488 lncRNAs, 33 miRNAs, and 1207 mRNAs with differentially expressed profiles. Cox proportional hazard analysis and survival analysis using the ceRNA network revealed four genes (IL‐11, PDGFD, NPTX1, ITPR1) as potential biomarkers of EAC prognosis in our predictor model, and IL‐11 was identified as an independent prognostic factor.

**Conclusions:**

In conclusion, we identified differences in the ceRNA regulatory networks and constructed a four–gene expression‐based survival predictor model, which could be referential for future clinical research.

## Graphical abstract

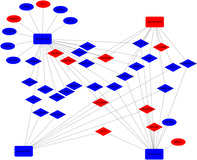

## Introduction

Cancer statistics in 2016 suggested that case mortality of esophageal cancer ranked sixth globally among all cancers, leading to an estimated 415 thousand deaths^1^ and a 20% five‐year survival rate.^2^ Despite recent developments in molecular‐targeted treatment and immunotherapy on the basis of surgery, chemotherapy and radiotherapy, these treatments have not yet shown a significant effect.^3^ Esophageal cancer includes adenocarcinoma and squamous cell carcinoma. The incidence of esophageal adenocarcinoma (EAC) has rapidly increased over the past decades and is resistant to current treatment with a poor prognosis.^4^ Identifying the biomarkers for its occurrence, development and prognosis is essential for understanding EAC and improving decisions in clinical practice.

Previous studies have suggested that the occurrence and development of cancers are regulated by both coding and noncoding RNAs with interaction between both.^5^, ^6^ LncRNA and miRNA are the two most‐studied noncoding RNAs.^7^ miRNAs are single‐stranded RNAs containing about 22 nucleotides that regulate protein transition through base‐pairing with mRNAs and inhibit translation of mRNAs.^8^, ^9^ LncRNAs are non‐coding RNAs containing more than 200 nucleotides that contribute to the regulation of epigenetics, cell cycle and cell differentiation.^10^ Both have been verified with recent application in the diagnosis and prognosis of various cancers.^11^, ^12^, ^13^

There have been previous efforts to identify biomarkers for the prognosis of EAC. Some have proposed a predictor model of EAC using miRNA,^14^, ^15^ while others have conducted prognostic risk factor analysis based on the interaction between miRNA and mRNA.^16^ However, to the best of our knowledge, there has not been any analysis using the lncRNA‐miRNA‐mRNA competing endogenous RNA (ceRNA) network for EAC, which provides a more reliable analysis.

In this study, we obtained transcriptome data of mRNA, miRNA and lncRNA from EAC patients and patients with normal esophageal mucosa as well as corresponding clinical information from TCGA. We analyzed the lncRNAs, miRNAs and mRNAs with different expressions and constructed a competing endogenous RNA (ceRNA) network. We then constructed a multivariate gene expression predictor model based on patient survival data and identified the independent prognostic factors. The results of the study contribute to the understanding of the underlying mechanism of EAC using ceRNA network and multigene predictor model, identify potential therapeutic and prognostic target genes and provide new directions for future research.

## Methods

### Patient datasets and data preprocessing

Sequencing data of the three types of RNAs in esophageal adenocarcinoma and normal esophageal tissues and their corresponding clinical data was obtained from The Cancer Genome Atlas (TCGA) database. Integration of this RNA data and extraction of the lncRNA expression profiles was done using the R bioconductor package TCGA Biolinks.^17^ Genes were annotated using the Ensembl online database (http://www.ensembl.org). LncRNA, miRNA, and mRNA expression profiles of the carcinoma and the normal were obtained. Our study was in compliance with the publication guidelines provided by TCGA, and the data obtained from TCGA did not require approval from the ethics committee.

### Differential expression analysis

The R Bioconductor package edgeR was employed to identify the differentially expressed lncRNAs (DE‐lncRNA), miRNAs (DE‐miRNA), and DEGs.^18^ Filtering criteria for the differential expression of the three RNAs in the normal and carcinoma groups were | fold change (logFC|) > 2 and adjust *P*‐value < 0.01 in DEGs and DE‐ lncRNAs and DE‐miRNAs. The corresponding heat map and clustering were created using the gplots package in R.

### Prediction of potential transcription factors, and target genes of DE‐miRNAs

The transcription factors of screened DE‐miRNAs were predicted using FunRich software.^18^ The screened upregulated and downregulated DE‐miRNAs were typed into this software. The top 10 predicted transcription factors are reported below.

### ceRNA network construction

LncRNA could regulate mRNA expression by acting as an miRNA sponge and contributing to ceRNA network. First, we decoded the miRNA sequences by using the starBase v2.0 database (http://starbase.sysu.edu.cn)^19^ and successfully paired DE‐miRNAs 3p or 5p transcript information. The miRcode database (http://www.mircode.org) and DIANA‐LncBase v2 were employed to construct lncRNA‐miRNA interaction pairs.miRDB (http://www.mirdb.org/), miRTarBase (http://mirtarbase.mbc.nctu.edu.tw/), and TargetScan (http://www.targetscan.org/) were used to predict target genes of the DE‐miRNAs and establish miRNA‐mRNA interaction pairs. The genes in all three databases are considered to be the target genes of the DE‐miRNAs. Using the Venny online website to compare the target genes, only the overlapping genes and their interaction pairs were further analyzed. Then, on account of the lncRNAmiRNA pairs and miRNA‐mRNA pairs, lncRNA‐miRNA‐mRNA ceRNA network was constructed using Cytoscape v3.6.1 software.^20^

### Functional enrichment analysis

GO, including biological processes (BP), molecular function (MF), cellular component (CC), is a commonly used approach for defining genes and its RNA or protein product to identify unique biological properties of highthroughput transcriptome or genome data.^21^ We used DAVID 6.8 (https://david.abcc.ncifcrf.gov/), an online bioinformatic tool designed to identify a large number of genes or proteins function^22^ to visualize the DEGs enrichment of GO (*P* < 0.05) and KEGG pathway analysis.

### Construction and validation of the **mRNA** risk score

A total of 76 patients were divided into two categories in a random manner: training dataset = 38, test dataset = 38. A training dataset was then analyzed to build an mRNA model that was later confirmed in the test and entire datasets. We used least absolute shrinkage and selection operator (LASSO) which is a generalized linear regression algorithm with simultaneous variable selection and regularization.^23^ An mRNA prognostic risk scoring model for survival prediction was constructed as follows:

$$\text{Risk score}=\sum_{i=1}^{n}\beta i*\text{gene}\;i$$

β indicates the coefficient of the mRNA, and gene represents mRNA expression value. The subjects were divided into low‐ and high‐risk groups according to the median score of the training dataset. Kaplan Meier (KM) and log‐rank methods were used to compare the survival rate between the groups. The time‐dependent receiver operating characteristic (ROC) curve was plotted by using the R “timeROC” package to evaluate specificity and sensitivity of the mRNA expression‐based prognostic signature. After that, the signature was confirmed in the test and entire datasets. ROC and KM curves were employed to verify the accuracy and feasibility of the mRNA model. All ROC and KM curves were plotted with R (version 3.5.3), and *P* < 0.05 represented statistical significance.

### Statistical analysis

Single variable Cox proportional risk (CPH) model was used to study the relationship between gene mRNA expression and survival. Based on KM analysis, gene mRNA expression and survival were entered in a multivariate CPH model. The results of CPH models are shown by HRs, and tests of significance coupled with 95% CI tests were carried out simultaneously. The classification of continuous variables which include risk score and RNAs expression value is specified in advance. *P* < 0.05 was considered statistically significant.

## Results

### **DEGs**, **DE‐lncRNAs**, **DE‐miRNAs** analysis

**Figure 1:**
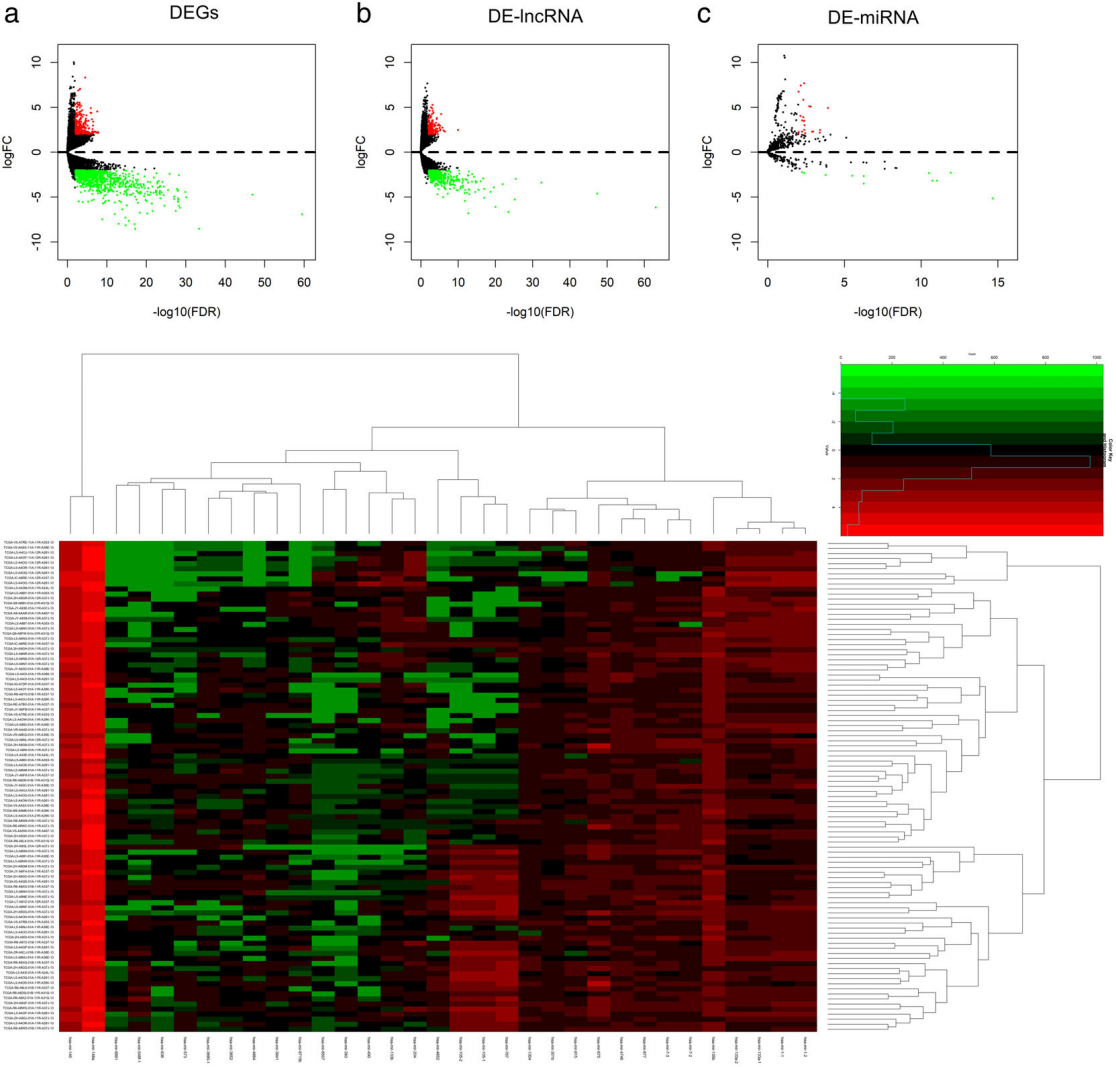
(**a**) DEGs; (**b**) DE‐lncRNA; and (**c**) DE‐miRNA are shown in the Volcano plot. Red node, upregulated miRNAs; green node, downregulated RNAs. (**d**) DE‐miRNA are shown in the heatmap.

We analyzed 78 esophageal adenocarcinoma tissues from EAC patients and nine normal tissues using data from TCGA database, and identified 1207 DEGs (Fig 1a) and 488 DE‐lncRNAs (Fig 1b). DEGs included 252 upregulated DEGs and 955 downregulated DEGs, and the DE‐lncRNA included 131 upregulated and 387 downregulated DEGs. We used the same methods and analyzed 88 EAC tissues and nine normal tissues and identified 33 DE‐miRNA (22 upregulated and 11 downregulated) (Fig 1c,d, Table 1).

**Table 1:** All 33 DE‐miRNAs included 22 upregulated and 11 downregulated miRNAs in the EAC tissues compared to normal esophageal tissues identified

DE‐miRNA	Name
Upregulated	hsa‐mir‐4664 hsa‐mir‐135b hsa‐mir‐4746 hsa‐mir‐877
	hsa‐mir‐301b hsa‐mir‐6715b hsa‐mir‐4652 hsa‐mir‐1304
	hsa‐mir‐3690‐1 hsa‐mir‐7‐3 hsa‐mir‐3652 hsa‐mir‐767
	hsa‐mir‐636 hsa‐mir‐548f‐1 hsa‐mir‐6891 hsa‐mir‐615
	hsa‐mir‐573 hsa‐mir‐105‐2 hsa‐mir‐675 hsa‐mir‐7‐2
	hsa‐mir‐3941 hsa‐mir‐105‐1
Downregulated	hsa‐mir‐490 hsa‐mir‐148a hsa‐mir‐133a‐2 hsa‐mir‐133a‐1
	hsa‐mir‐145 hsa‐mir‐204 hsa‐mir‐1‐2 hsa‐mir‐1‐1 hsa‐mir‐133b
	hsa‐mir‐383 hsa‐mir‐6507

### Prediction of upregulated and downregulated transcription factors of **DE‐miRNAs**

**Figure 2:**
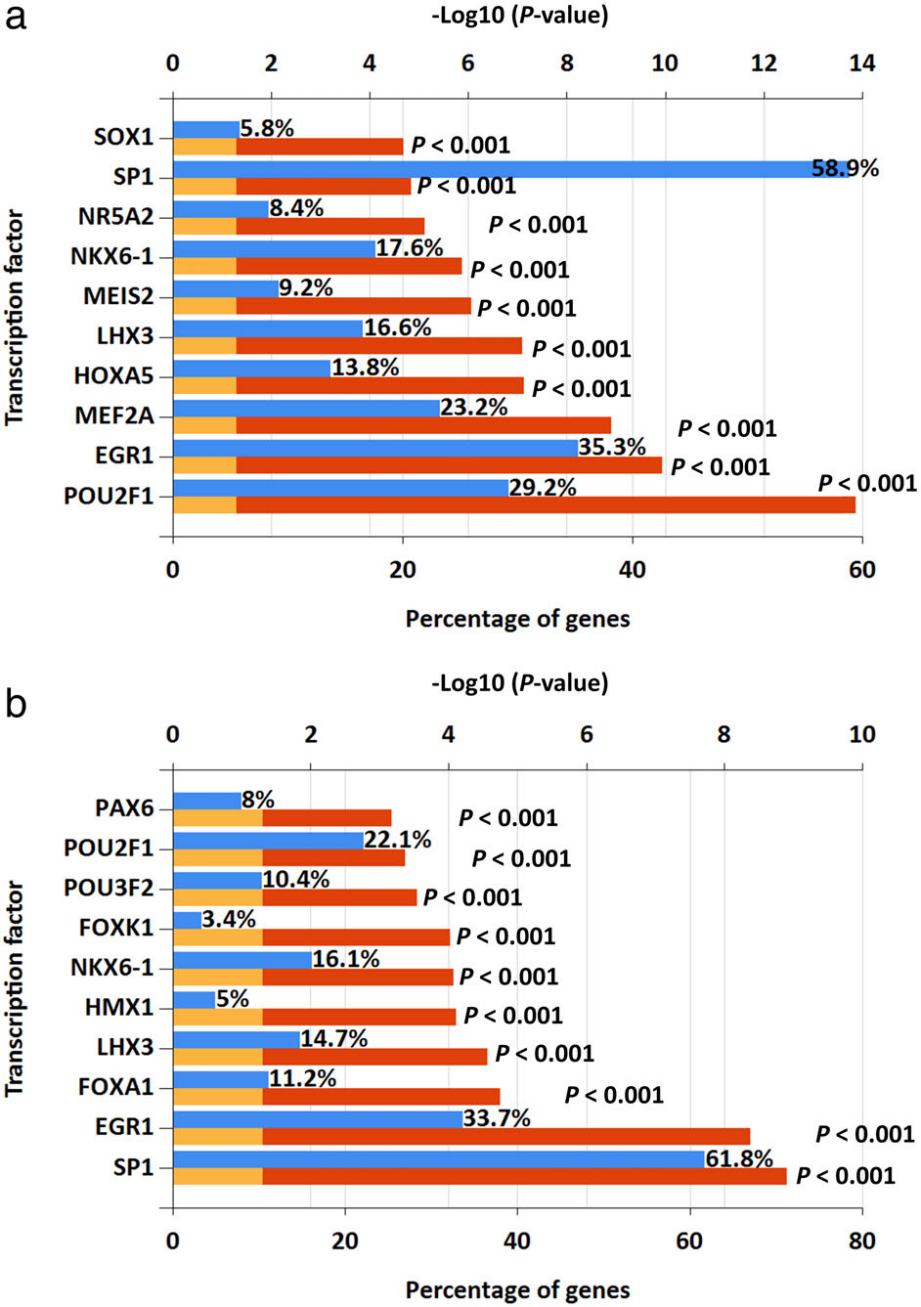
Predicted transcription factors of DE‐miRNAs. (**a**) Transcription factors of upregulated DE‐miRNAs (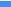) Percentage of gene (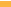) *P* = 0.05 (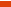) *P*‐value; (**b**) transcription factors of downregulated DE‐miRNAs (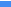) Percentage of gene (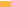) *P* = 0.05 (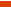) *P*‐value.

We used FunRich software to predict the transcription factors of up‐ and downregulated DE‐miRNA (Fig 2a,b). The top 10 transcription factors for upregulated transcription factors of DE‐miRNAs were SOX1, SP1, NR5A2, NKX6‐1, MEIS2, LHX3, HOXA5, MEF2A, EGR1, POU2F1, while the top 10 for downregulated transcription factors were PAX6, POU2F1, POU3F2, FOXK1, NKX6‐1, HMX1, LHX3, FOXA1, EGR1, SP1.

### Construction of **ceRNA** network in **EAC**

**Figure 3:**
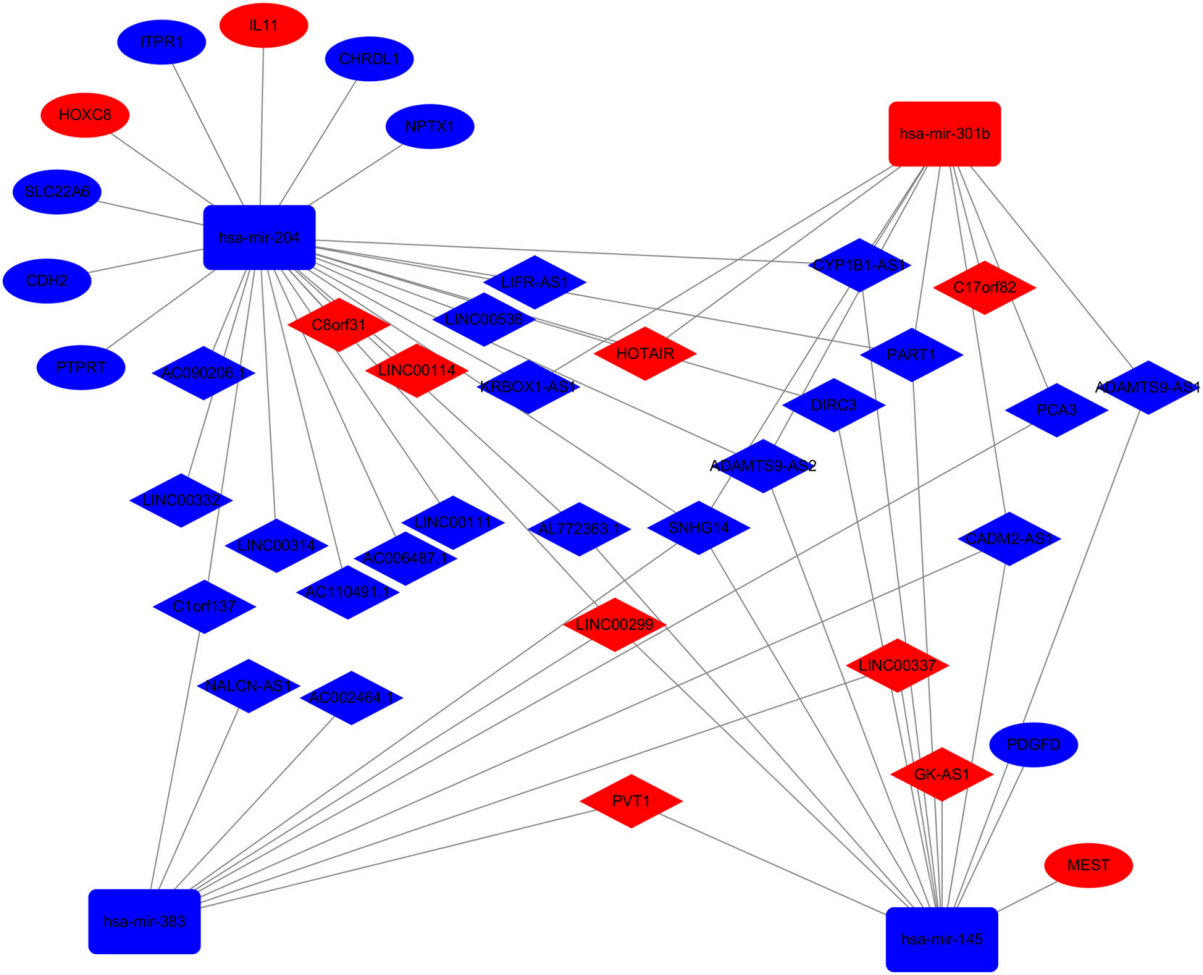
ceRNA networks of EAC. Red represents upregulation, and blue represents downregulation. lncRNAs, miRNAs, and mRNAs in the networks are represented as diamonds, round rectangles, and circles, respectively.

Based on the lncRNA‐miRNA and miRNA‐mRNA pairs, we constructed and visualized the ceRNA network graph using Cytoscape v3.6.1 (Fig 3). We identified 43 common RNAs (29 lncRNAs, four miRNAs, and 10 mRNAs) in the ceRNA network. The node connections in the network represented the interactions between RNAs, and the RNAs with more important biological functions were those with stronger connectivity in the graph. We then included 10 mRNAs (NPTX1, CDH2, ITR1, PDGFD, SLC22A6, MEST, IL11, CHRDL1, PTPRT, and HOXC8) for further analysis.

### **GO** and **KEGG** pathway analysis

**Figure 4:**
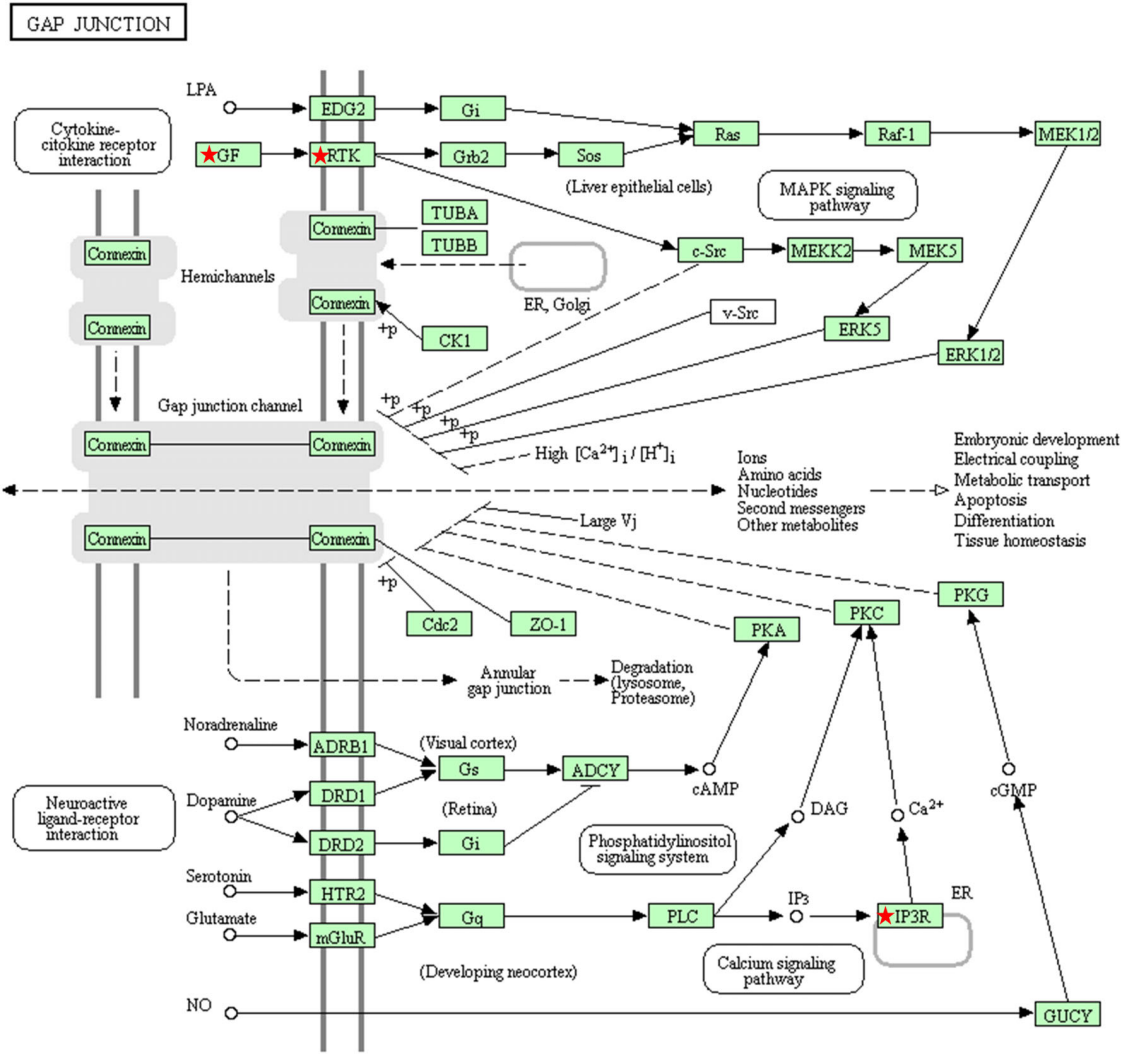
The most significant KEGG pathway of the 10 mRNAs. GF: growth factor; IP3R: inositol 1,4,5‐trisphosphate receptor; RTK: receptor tyrosine kinases.

We performed gene ontology (GO) and KEGG pathway analysis for the 10 mRNAs identified in the last step using Enrichr software. The results, as shown in Table 2, suggested that biological process (BP) included homophilic cell adhesion, megakaryocyte differentiation, central nervous system development, urate transport, secretion, etc; cell components (CC) included endoplasmic reticulum lumen, platelet dense granule membrane, platelet dense granule, catenin complex, sarcoplasmic reticulum, etc, and molecular functions (MF) included growth factor receptor binding, salt and urate transmembrane transporter activity, calcium channel inhibitor activity, etc. KEGG pathway analysis identified five pathways unique to the EAC group, including cytokine‐cytokine receptor interaction, MAPK signaling, calcium signaling, phosphatidylinositol signaling system, and neuroactive ligand‐receptor interaction, all of which belonged to gap junction (Fig 4 & Table 3).

**Table 2:** Significantly enriched gene ontology (GO) biological process terms

	Term	*P*‐value Odds ratio Combined score		
GOTERM_BP_DIRECT	Homophilic cell adhesion via plasma membrane adhesion molecules (GO:0007156)	4.46E‐04	62.5	482.175 804 5
GOTERM_BP_DIRECT	Cell‐cell adhesion via plasma membrane adhesion molecules (GO:0098742)	0.00217	28.169 01	172.761 874 8
GOTERM_BP_DIRECT	Megakaryocyte differentiation (GO:0030219)	0.00449	222.2222	1201.220 433
GOTERM_BP_DIRECT	Central nervous system development (GO:0007417)	0.00498	18.433 18	97.741 405 06
GOTERM_BP_DIRECT	Urate transport (GO:0015747)	0.00499	200	1060.071173
GOTERM_BP_DIRECT	Regulation of hormone secretion (GO:0046883)	0.00599	166.6667	853.0805526
GOTERM_BP_DIRECT	Secretion (GO:0046903)	0.00648	153.8462	775.179 255 7
GOTERM_BP_DIRECT	negative regulation of hormone secretion (GO:0046888)	0.00648	153.8462	775.179 255 7
GOTERM_BP_DIRECT	Gliogenesis (GO:0042063)	0.00698	142.8571	709.254 538 4
GOTERM_BP_DIRECT	Platelet‐derived growth factor receptor signaling pathway (GO:0048008)	0.00847	117.6471	561.329 363 8
GOTERM_CC_DIRECT	Endoplasmic reticulum lumen (GO:0005788)	2.72E‐04	22.222 22	182.423 328 4
GOTERM_CC_DIRECT	Platelet dense granule membrane (GO:0031088)	0.003	333.3333	1936.761 417
GOTERM_CC_DIRECT	Platelet dense granule (GO:0042827)	0.01045	95.2381	434.370 388 2
GOTERM_CC_DIRECT	Catenin complex (GO:0016342)	0.01392	71.428 57	305.341 398 5
GOTERM_CC_DIRECT	Sarcoplasmic reticulum (GO:0016529)	0.01441	68.965 52	292.407 786 6
GOTERM_CC_DIRECT	Sarcoplasm (GO:0016528)	0.0154	64.516 13	269.269 084 6
GOTERM_CC_DIRECT	Intercalated disc (GO:0014704)	0.0154	64.516 13	269.269 084 6
GOTERM_CC_DIRECT	Cortical actin cytoskeleton (GO:0030864)	0.0257	38.461 54	140.812 704 8
GOTERM_CC_DIRECT	Cortical cytoskeleton (GO:0030863)	0.0257	38.461 54	140.812 704 8
GOTERM_CC_DIRECT	Cytoplasmic vesicle membrane (GO:0030659)	0.02668	37.03704	134.216 258 4
GOTERM_MF_DIRECT	Growth factor receptor binding (GO:0070851)	9.20E‐04	43.478 26	303.984 707 9
GOTERM_MF_DIRECT	Salt transmembrane transporter activity (GO:1901702)	0.00449	222.2222	1201.220 433
GOTERM_MF_DIRECT	Urate transmembrane transporter activity (GO:0015143)	0.00449	222.2222	1201.220 433
GOTERM_MF_DIRECT	Calcium channel inhibitor activity (GO:0019855)	0.00499	200	1060.071173
GOTERM_MF_DIRECT	Inositol 1,4,5 trisphosphate binding (GO:0070679)	0.00549	181.8182	946.412 758
GOTERM_MF_DIRECT	Platelet‐derivedgrowth factor receptor binding (GO:0005161)	0.00648	153.8462	775.179 255 7
GOTERM_MF_DIRECT	Intracellular ligand‐gated ion channel activity (GO:0005217)	0.00748	133.3333	652.801 799 4
GOTERM_MF_DIRECT	Calcium ion binding (GO:0005509)	0.00839	14.08451	67.339 577 8
GOTERM_MF_DIRECT	Organic acid transmembrane transporter activity (GO:0005342)	0.00847	117.6471	561.329 363 8
GOTERM_MF_DIRECT	Ion antiporter activity (GO:0099516)	0.00897	111.1111	523.818 423 7

**Table 3:** Significant KEGG pathways

Category	Term	*P*‐value	Pop Hits	Pop Total	Fold Enrichment	FDR
KEGG_pathway	bta04540:Gap junction	0.035	89	7550	42.416	28.187

### Cox proportional hazard analysis

**Figure 5:**
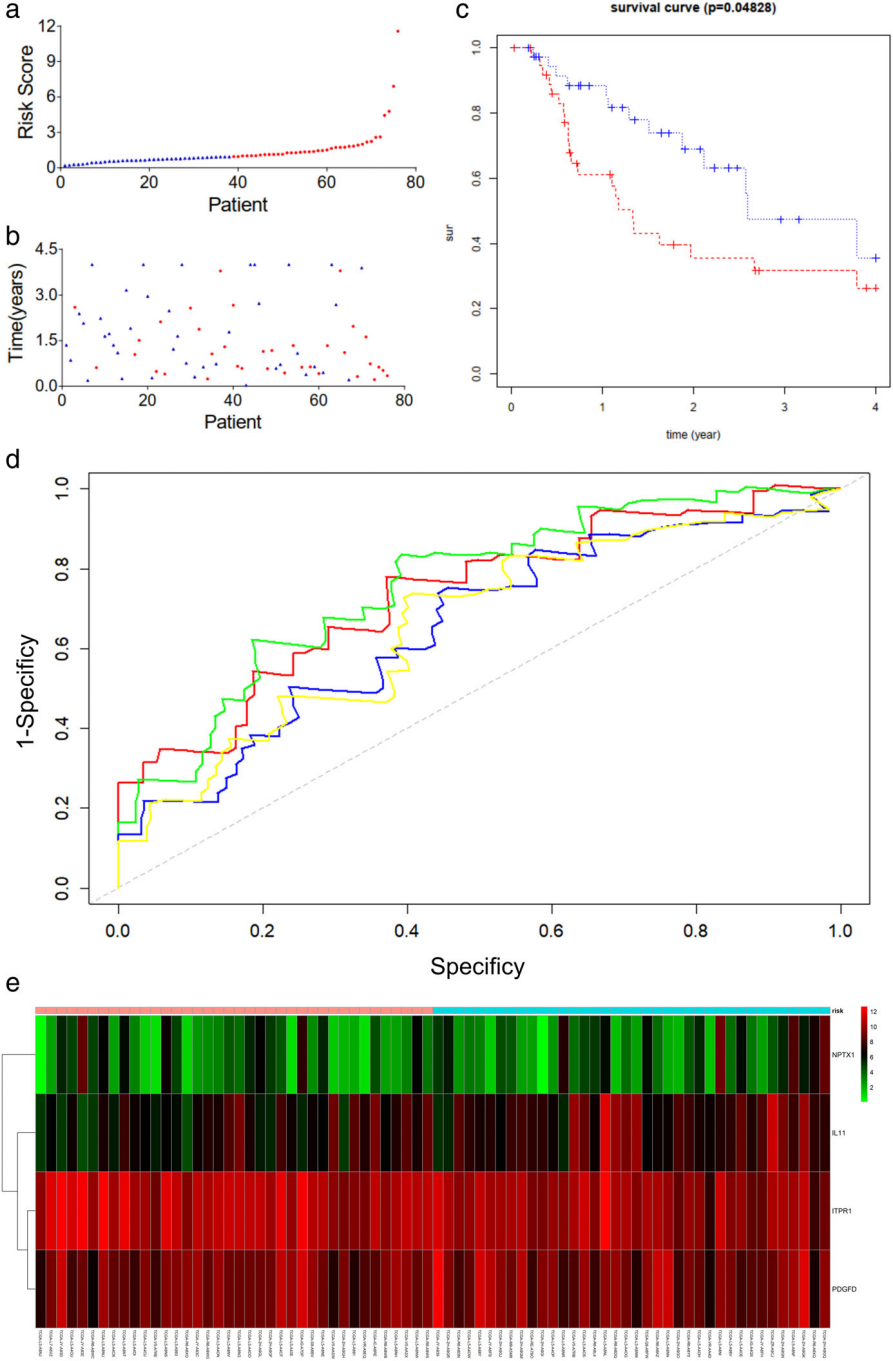
The four mRNA signature predicted the OS of EAC patients in the test and entire datasets. The mRNA signature risk score distribution and heatmap of the mRNA expression profiles in the test dataset (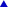) low risk (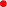) high risk (**a**, **b**, and **e**) (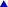) high (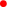) low. Survival curves of high‐ and low‐risk samples in the test dataset (**c**) (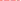) high risk (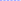) low risk. Time‐dependent ROC curve for accuracy of the predicting risk score system in the test dataset (**d**) (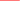) 1 years 0.735 (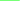) 2 years 0.759 (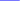) 3 years 0.656 (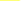) 4 years 0.662.

We also calculated a risk score for each subject based on the status of four genes using a Cox regression model (Table 4): coxph(formula = Surv(futime, fustat) ~ NPTX1 + ITPR1 + PDGFD + IL11, data = rt). We further stratified EAC patients into high‐ and low‐risk groups and found that four‐year survival rate was 25% and 35% for high‐ and low‐risk patients, respectively (Tables 5 and 6). We then ranked the risk scores and visualized the survival status of each patients using a dotplot. Mortality rate of patients was lower for patients in the low‐risk group in comparison to the high‐risk group (Fig 5a,b). Survival time of high‐risk patients was shorter than low‐risk patients according to the Kaplan‐Meier curve (Fig 5c). Using a time‐dependent ROC curve, we described the predictive value of the proposed four gene expression predictor model, and the area under curve (AUC) values were 0.735, 0.759, 0.656, 0.662 at one, two, three and four years of the four‐gene signature (Fig 5d). The four‐gene profile heatmap is shown in Fig 5e.

**Table 4:** Four genes Cox regression model

	coef	exp(coef)	se(coef)	z	*P*
NPTX1	0.216 04	1.241 15	0.09697	2.228	0.02589
ITPR1	−0.7486	0.473 01	0.234 71	−3.190	0.00142
PDGFD	0.352 19	1.422 18	0.199 50	1.765	0.07750
IL11	0.265 45	1.304 02	0.114 78	2.313	0.02074

Likelihood ratio test = 13.6 on 4 df, *P* = 0.008682, *n* = 76, number of events = 38.

**Table 5:** High‐risk EAC patients with statistically significant influence in survival curve

Time	Risk (n)	Event (n)	Survival	Standarderror	Lower 95% CI	Upper 95% CI
0.219	36	1	0.972	0.0274	0.92	1
0.318	35	1	0.944	0.0387	0.873	1
0.345	34	1	0.917	0.0461	0.831	1
0.416	32	1	0.888	0.0528	0.790	0.998
0.436	31	1	0.859	0.0583	0.752	0.982
0.521	29	1	0.830	0.0634	0.714	0.964
0.575	28	1	0.800	0.0677	0.678	0.944
0.586	27	1	0.770	0.0714	0.643	0.924
0.625	25	1	0.740	0.0749	0.607	0.902
0.627	24	1	0.709	0.0779	0.572	0.879
0.638	23	1	0.678	0.0803	0.537	0.855
0.658	21	1	0.646	0.0828	0.502	0.830
0.734	19	1	0.612	0.0851	0.466	0.803
1.107	17	1	0.576	0.0874	0.428	0.775
1.145	16	1	0.540	0.0890	0.391	0.746
1.175	15	1	0.504	0.0901	0.355	0.715
1.334	14	1	0.468	0.0905	0.320	0.684
1.34	13	1	0.458	0.0908	0.311	0.651
1.622	12	1	0.396	0.0898	0.254	0.617
1.973	10	1	0.356	0.0891	0.218	0.582
2.666	9	1	0.317	0.875	0.184	0.544
3.797	6	1	0.258	0.0863	0.134	0.505

**Table 6:** Low‐risk EAC patients with statistically significant influence in survival curve

Time	Risk (n)	Event (n)	Survival	Standarderror	Lower 95% CI	Upper 95% CI
0.238	37	1	0.973	0.0267	0.922	1
0.403	33	1	0.943	0.0389	0.870	1
0.488	32	1	0.914	0.0475	0.825	1
0.611	31	1	0.885	0.0544	0.784	0.998
1.044	26	1	0.851	0.0620	0.737	0.981
1.063	25	1	0.816	0.0682	0.693	0.962
1.296	22	1	0.779	0.0746	0.646	0.940
1.507	19	1	0.738	0.0811	0. 595	0.916
1.874	15	1	0.689	0.0894	0.534	0.889
2.118	12	1	0.632	0.0987	0.465	0.858
2.570	8	1	0.553	0.1136	0.369	0.827
2.595	7	1	0.474	0.1218	0.286	0.784
3.789	4	1	0.355	0.1373	0.167	0.758

### Kaplan–Meier analysis of the four‐gene expression predictor model

**Figure 6:**
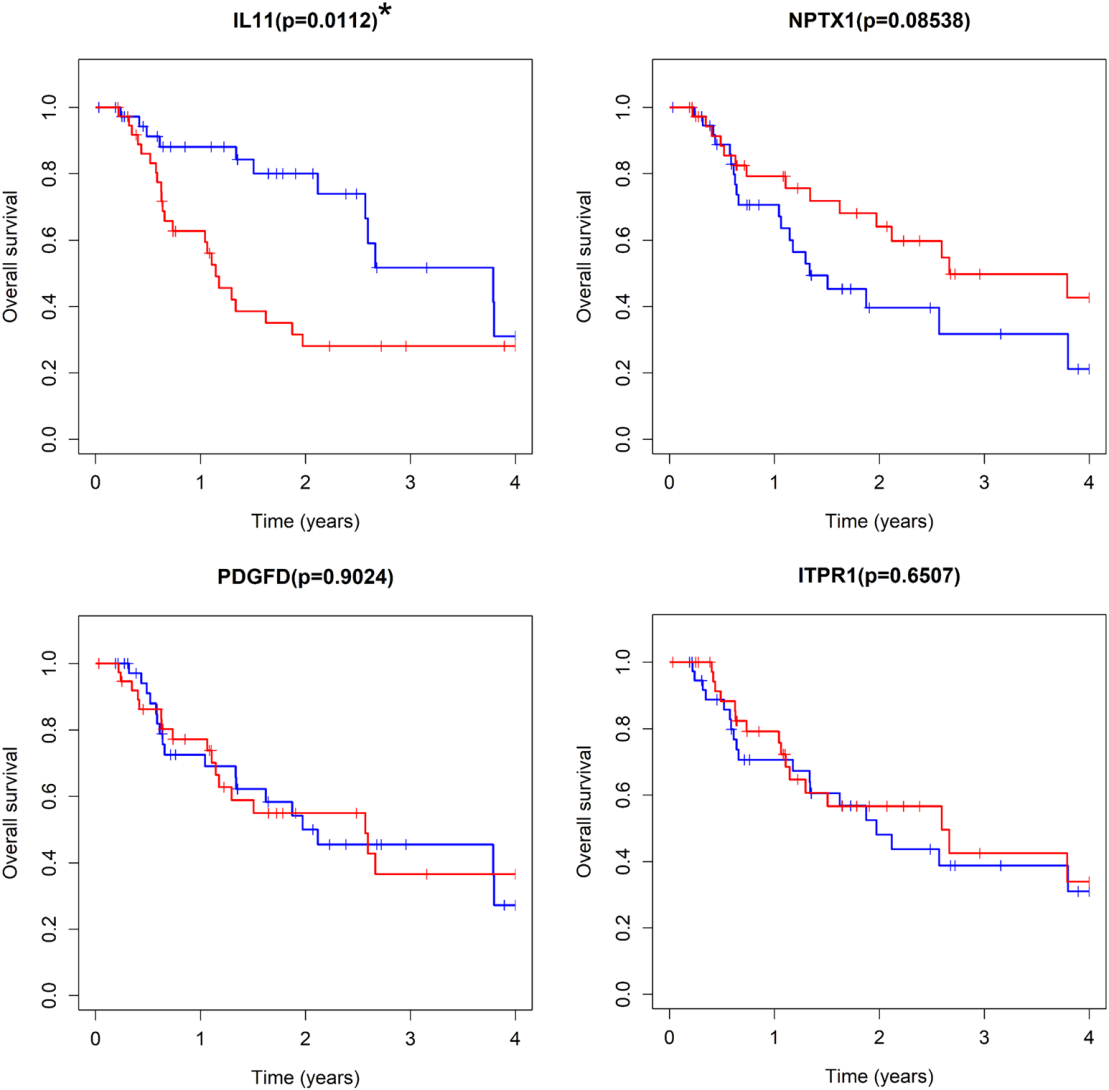
Stratified analysis of overall survival in the entire dataset. Kaplan–Meier analysis for OS by IL‐11, NPTX1, PDGFD, and ITPR1 mRNAs. *<0.05. (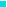) low expression (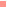) high expression. (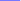) low expression (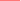) high expression. (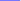) low expression (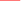) high expression. (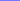) low expression (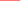) high expression.

Last but not least, we assessed the prognostic value of the four‐gene expression model using R and found that increased expression of *IL11* was associated with worse overall survival (*P* < 0.05) while the low expression of *NPTX1*, *ITPR1*, and *PDGFD* were not (Fig 6). *IL‐11* was upregulated in EAC tissues, while *NPTX1*, *ITPR1* and *PDGFD* were downregulated in EAC tissues.

## Discussion

Morbidity and mortality of EAC have not significantly improved despite progress in its pathogenesis and clinical treatment, due to the lack of reliable biomarkers and specific genes to guide individualized treatment.^24^ Research on molecular markers of EAC is urgently needed to customize effective individualization of treatment and improve survival. Sequencing technology and bioinformatics allow the screening of the entire DNA mutation profile and contribute to the understanding of EAC origin and characteristics. However, screening biomarkers using changes in mRNAs only are insufficient as miRNAs, lncRNAs and the interaction between these three all play important roles in the growth and differentiation of cells and occurrence of cancers.^25^, ^26^ ceRNA hypothesis provides an in‐depth understanding of the mechanism of carcinogenesis and new evidence for cancer diagnosis and treatment, chemotherapy efficacy prediction and cancer risk prediction.^6^, ^27^, ^28^

Data mining using TCGA database is an effective approach to identify changes in RNA expression that are relevant to clinical outcomes and to screen new therapeutic targets. While ceRNAs have been studied more in the past decades, few studies have evaluated the prognostic value of ceRNA for EAC patients using TCGA database. Here, in the ceRNA network, we identified 29 lncRNAs, four miRNAs and 10 mRNAs that were significantly different between EAC and normal patients using TCGA, and only two of the 10 mRNAs have been previously reported (CDH2^29^ and PDGFD^30^). Based on the survival data up to 2017, we constructed a four‐gene expression predictor model (*IL‐11*, *NPTX1*, *ITPR1*, *PDGFD*) using multivariate Cox regression analysis and also conducted univariate analyses individually for the four genes. Our results showed that IL‐11 could potentially be used as an independent prognostic factor for EAC. Among the interleukin family, secretion of IL‐33 in esophageal epithelial cells has been reported to prompt the occurrence of gastroesophageal reflux diseases and lead to Barrett esophagus;^31^ IL‐4, IL‐13,^32^ IL‐1β^33^ and IL‐6 ^34^ contributed to increased secretion of esophageal mucosa in patients with Barrett's esophagus, and IL‐11 contributed to esophageal squamous cell carcinoma progression and its aggressiveness.^35^ Nonetheless, to date, no study has reported the role of IL‐11 in EAC, and our study fills this gap and confirms the potentially important role of it in EAC prognosis.

IL‐11 is a member of the IL‐6 family of cytokines,^36^ and has been recognized for its role in the disease pathogenesis of mucosal homeostasis including gastrointestinal cancers.^37^ IL‐11 could increase the tumorigenic abilities of cells including the survival of cells or origin, proliferation of cancer cells, and survival of metastatic cells of distant organs.^38^, ^39^ Recent studies have also suggested its role in osteosarcoma deterioration,^40^ postoperative recurrence of liver cancer^41^, ^42^ and migration and survival of gastric cancer cells.^43^ Based on the current knowledge of the biological properties of IL‐11 and its role in cancer, IL‐11 signaling inhibition might be a new therapeutic approach for cancer treatment.^44^ In pancreatic ductal adenocarcinoma,^45^ lung adenocarcinoma,^46^, ^47^ gastric adenocarcinoma,^48^ and colorectal adenocarcinoma, IL‐11‐STAT3 signaling induced invasion and enhanced development of adenocarcinoma. In addition, IL‐6/11 is one of the highly specific biomarkers with great accuracy for the diagnosis of lung adenocarcinoma in bronchoalveolar lavage fluid specimens.^49^ The molecular mechanism of IL‐11 in esophageal adenocarcinoma is the focus of our future research.

NPTX1 is a member of the pentraxin family and a major risk factor for nervous system disorders,^50^ and its downregulation expression has been reported in many cancers.^51^, ^52^, ^53^, ^54^ Its function in EAC is still unclear and some studies have suggested it plays a role in regulating cell migration^55^ and tumor metastasis.^56^ ITPR1 is considered as the most prominent gene in regulating cancer cell resistance to NK‐mediated lysis^57^ and has been shown to be involved in the regulation of intracellular calcium signaling and the regulation of autophagy.^58^ in vivo studies have previously indicated that ITPR1 targeting in cancer cells in combination with NK depletion contributed to tumor growth, indicating its role in the regulation of in vivo susceptibility of renal carcinoma cells to NK activities.^57^ These studies suggest a putative role of ITPR1 in cancer progression and immune resistance. PDFD‐D plays an important role in cell proliferation, apoptosis, transformation, invasion, metastasis, angiogenesis and other biological processes,^59^ and its downregulation expression has been reported to inhibit the NF‐κB pathway for cell proliferation and invasion, and induce apoptosis in esophageal squamous cell carcinoma.^30^ It contributes to ibrutinib resistance of diffuse large B‐cell lymphoma through EGFR activation^60^ and also the prognosis of gastric adenocarcinoma patients.^61^

There have been many efforts to identify biomarkers for EAC prognosis. Lan *et al*. reported a six miRNA signature of esophageal adenocarcinoma in 2019.^14^ Skinner* et al*. reported a validated miRNA profile to predict response to therapy in esophageal adenocarcinoma.^15^ However, they focused on miRNA while in our study we focused on mRNA, and the results of part of the six miRNA are consistent with those involved in our study. Our results remain in general agreement with the previous conclusions. Also, Dong *et al*. reported five genes that have a connection with overall or relapse‐free survival.^16^ However, the credibility of the result which shows the identification of DEGs in mRNA expression profiling data sets GSE1420, GSE26886, and GSE92396 was not high as the three data sets come from different platforms (GSE1420 from GPL96, GSE26886 from GPL570, GSE92396 from GPL6244).

In addition, EAC is extremely rare in China, and we made every attempt to contact many hospitals and research institutes during the study period. Although a small number of survival data are available, we have still been unable to find enough pathological specimens of this kind for further measurement (eg, PCR, next‐generation sequencing, and sanger sequencing). We therefore divided the patients into two categories in a random manner: training dataset and test dataset, thereby hoping to minimize bias.

In conclusion, we constructed a ceRNA network and a four‐gene expression predictor model using data from TCGA, a large‐scale sequence database, that could be used to perform comprehensive multidimensional analysis. However, future studies are needed to validate our findings considering the complex interaction of the network.

## Disclosure

No conflict of interest is reported.
